# Testing the John Henryism Hypothesis on Cardiometabolic Health Among Older African Americans

**DOI:** 10.1093/geroni/igab046.1547

**Published:** 2021-12-17

**Authors:** Yanping Jiang, Jennifer Gómez, Jacqueline Rodriguez-Stanley, Samuele Zilioli

**Affiliations:** 1 Rutgers University, New Jersey, United States; 2 Wayne State University, Wayne State University, Michigan, United States; 3 Wayne State University, Detroit, Michigan, United States

## Abstract

In the context of racism, the John Henryism Hypothesis posits that prolonged high-effort coping, which is referred to as John Henryism, may take a toll on physical health among individuals from low socioeconomic status (SES) backgrounds, particularly low SES African Americans. This study aimed to test the John Henryism Hypothesis among older African Americans by examining the combined effect of John Henryism and childhood SES on cardiometabolic health indexed by metabolic syndrome and systemic inflammation. Data were drawn from a sample of 170 urban older African Americans (Mage = 67.4 years, 75.9% female), who completed questionnaires assessing John Henryism and childhood SES (i.e., parental education). Blood pressure, waist circumference, and fasting blood were also collected to assess metabolic syndrome and systemic inflammation. Results indicated that John Henryism was significantly associated with elevated metabolic syndrome symptoms among older African Americans reporting low childhood SES (b = 0.42, 95%CI = [0.02, 0.83]), but not among those with high childhood SES (b = -0.33, 95%CI = [-0.78, 0.13]). This result was robust to a variety of demographic variables, lifestyle behavioral factors, and health conditions that are known to be associated with metabolic syndrome. A similar pattern of results, however, did not emerge for systemic inflammation. Our findings highlight the importance of considering the joint impact of early childhood socioeconomic backgrounds and individual psychological proclivities in explaining the elevated cardiovascular disease risk among older African Americans.

